# Amyloid β42 peptide is toxic to non-neural cells in *Drosophila* yielding a characteristic metabolite profile and the effect can be suppressed by PI3K

**DOI:** 10.1242/bio.029991

**Published:** 2017-11-15

**Authors:** Mercedes Arnés, Sergio Casas-Tintó, Anders Malmendal, Alberto Ferrús

**Affiliations:** 1 Dept. of Molecular, Cellular and Developmental Neurobiology, Instituto Cajal, Avda. Doctor Arce, 37, 28002 Madrid, Spain; 2 Biochemistry and Structural Biology, Center for Molecular Protein Science, Department of Chemistry, Lund University, P.O. Box 124, SE-22100 Lund, Sweden

**Keywords:** Amyloid β, Metabolomics, NMR, PI3K, Epithelial cells, Wingless

## Abstract

The human Aβ42 peptide is associated with Alzheimer's disease through its deleterious effects in neurons. Expressing the human peptide in adult *Drosophila* in a tissue- and time-controlled manner, we show that Aβ42 is also toxic in non-neural cells, neurosecretory and epithelial cell types in particular. This form of toxicity includes the aberrant signaling by Wingless morphogen leading to the eventual activation of Caspase 3. Preventing Caspase 3 activation by means of p53 keeps epithelial cells from elimination but maintains the Aβ42 toxicity yielding more severe deleterious effects to the organism. Metabolic profiling by nuclear magnetic resonance (NMR) of adult flies at selected ages post Aβ42 expression onset reveals characteristic changes in metabolites as early markers of the pathological process. All morphological and most metabolic features of Aβ42 toxicity can be suppressed by the joint overexpression of PI3K.

## Abstract (precis)

**Summary:** The Alzheimer's disease-related Aβ42 peptide is toxic for non-neural cells. This toxicity can be detected by specific metabolite changes and suppressed by the overexpression of the enzyme PI3K.

## INTRODUCTION

Aβ42 is a proteolytic peptide from the amyloid precursor protein (APP), a ubiquitous transmembrane protein whose physiological function is still poorly characterized (Ludewig and Korte, 2016). APP proteolysis can produce two different peptides, Aβ40 and Aβ42, which accumulate inside and outside the cell where they may form amyloid fibrils (Gerber et al., 2017; Seipold and Saftig, 2016; Bolduc et al., 2016; Andrew et al., 2016). The second, Aβ42, is more toxic and seems to be the origin of Alzheimer’s disease (AD). The toxicity mechanisms are still largely unknown, but current views indicate that Aβ monomers and fibrils are relatively inert. By contrast, smaller oligomeric aggregates, formed on the pathway between monomer and fibril, are the neurotoxic molecule (Walsh et al., 2002; Lei et al., 2016).

Since AD was first described as a neural disease, the vast majority of studies on Aβ42 have focused on neurons. This is in spite of early evidence reporting the expression of APP in non-neuronal cells and the secretion of its Aβ peptides (Card et al., 1988; Busciglio et al., 1993). AD-hallmark proteins have been reported in patients diagnosed with other diseases, such as sporadic inclusion body myositis (Askanas and Engel, 1998) or several forms of autism (Westmark et al., 2016). In the absence of effective treatments for fully developed AD, the only option is to explore procedures for an early diagnosis. In this context, metabolic alterations are part of neurodegenerative disorders, including AD, as supported by studies in animal models and clinical samples from AD patients (reviewed in Barba et al., 2008; Trushina et al., 2013). Potential biomarkers of AD include n-acetylaspartate as a neuronal loss marker, and myo-inositol for gliosis and inflammation. Recently, a 12 plasma metabolite profile specific to superior memory performance in older adults was identified which allowed prediction of which patients with mild cognitive impairment will progress to AD (Mapstone et al., 2017).

Metabolic profiling can be performed by nuclear magnetic resonance (NMR) or mass spectrometry (MS). Perturbations often begin with metabolic changes, hence metabolite profiling by NMR is a good starting point for an early diagnosis, perhaps to be followed by recently developed MS procedures to detect Aβ42 and tau if their discriminatory value for AD versus other diseases is finally demonstrated (Pottiez et al., 2017; Somers et al., 2017). Here, we use *Drosophila* to reproduce Aβ42 accumulation and to assay toxicity suppression methods. In particular, we focus on PI3K given its neuroprotective effects (Martin-Pena et al., 2006; Sofola et al., 2010; Cuesto et al., 2011, 2015). Our study focuses on the much-neglected non-neuronal cell types as a strategy towards their potential use in early diagnosis of AD.

## RESULTS AND DISCUSSION

**Fig. 1.**
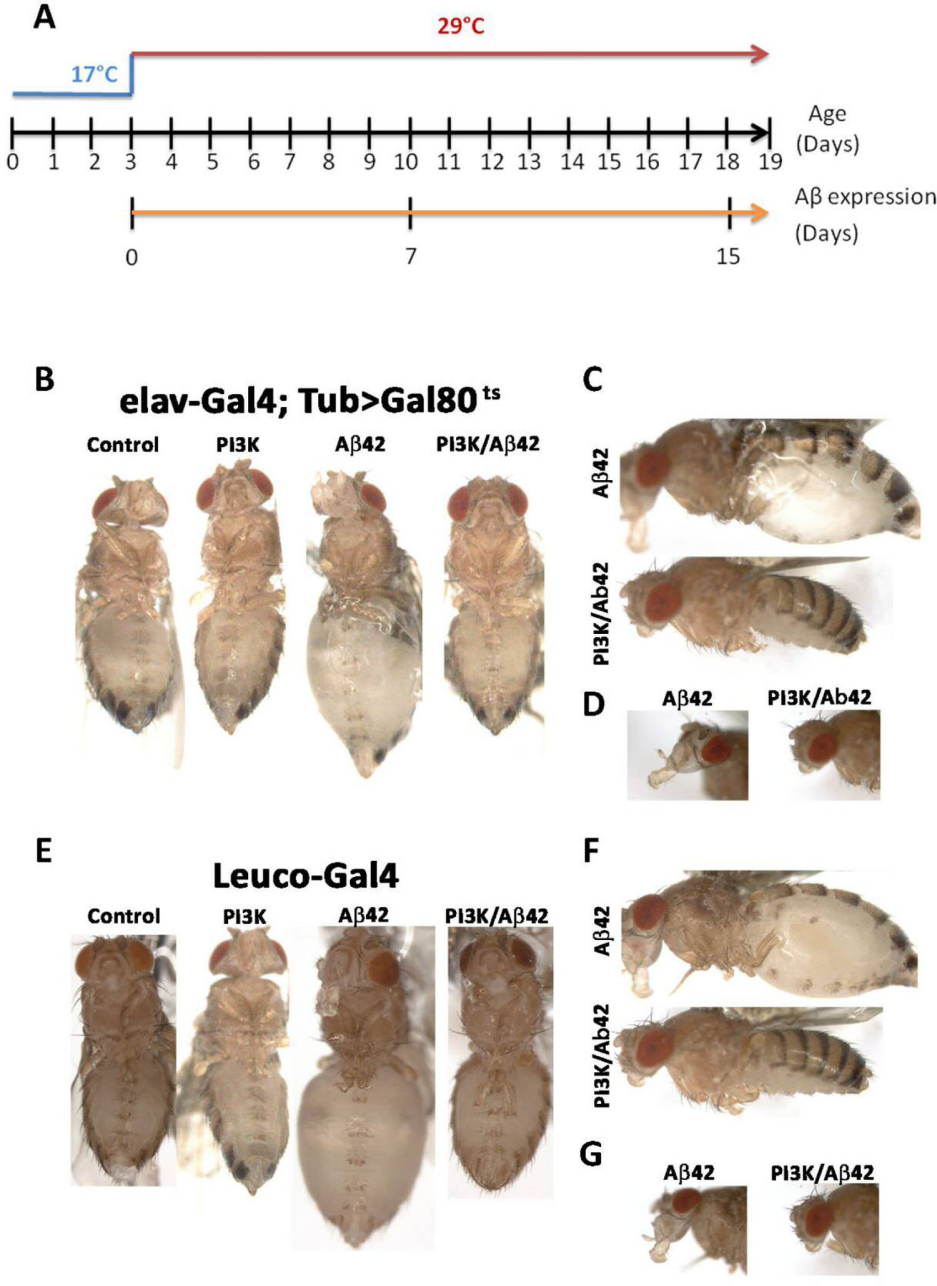
**The inflated abdomen phenotype induced by Aβ42 is prevented by PI3K overexpression.** (A) Diagram of the temperature shift schedule to inactivate the *Gal80^TS^* repressor, thus allowing UAS-constructs expression. System onset was routinely established at day 0-3 after eclosion and the effects were monitored 7 or 15 days later. (B) Representative images of 15-day-old adult flies expressing: *UAS-LacZ* (control), *UAS-Aβ42(2x), UAS-PI3K^CAAX^* and *UAS-PI3K^CAAX^/UAS-Aβ42(2x)* under *elav^C155^-Gal4/Tub-Gal80^TS^* driver. (C) Lateral views of Aβ42 and PI3K/Aβ42 genotypes. (D) Lateral views of head and proboscis of Aβ42 and PI3K/Aβ42. (E) Representative images of 15-day-old adult flies expressing: *UAS-LacZ* (control), *UAS-Aβ42(2x), UAS-PI3K^CAAX^* and *UAS-PI3K^CAAX^/UAS-Aβ42(2x)* under the *Leuco-Gal4* driver. (F,G) Lateral views of whole body (F) or head-proboscis (G) of Aβ42 and PI3K/Aβ42 flies.

We drive the expression of a construct with two copies of the human Aβ42 peptide encoding gene (Casas-Tintó et al., 2011) using the binary system Gal4/UAS (Brand and Perrimon, 1993) coupled with the temperature-sensitive repressor Gal80^ts^ (McGuire et al., 2003). The time onset was routinely established at day 0-3 of adulthood and the effects were monitored 7 and 15 days later (Fig. 1A).

### Aβ42 accumulation induces toxicity in neurosecretory cells

Driving Aβ42 to the nervous system (*elav-Gal4*), from adult day 0-3 onwards (see genotypes in the legend of Fig. 1), all flies exhibit inflated abdomen and proboscis, up to 80% of their normal width, by day 7-10 (Fig. 1B-D). Upon puncture, the inflated structures expelled abundant liquid which suggested a problem with diuresis. To explore this suggestion, we repeated the experiment driving the expression of Aβ42 to the neurosecretory cells that express the peptide Leucokinin (*leuco-Gal4*). This peptide controls food intake and fluid secretion through the Malpighian tubules (Terhzaz et al., 1999; Al-Anzi et al., 2010; López-Arias et al., 2011). The inflated abdomen phenotype was reproduced (Fig. 1E-G). The phenotype was evident also from the first days of adulthood and continued increasing its severity until death. This feature represents Aβ42 toxicity in cells outside the neuron type.

In a previous study, we had shown that overexpression of PI3K delays the deleterious features of aged neurons (Martin-Pena et al., 2006). To assay the possible counter-effect of PI3K upon this non-neural toxicity, we co-expressed Aβ42 and a constitutive active form of PI3K, PI3K^CAAX^, in the *leuco-Gal4* domain. In 100% of individuals (*n*=46), the inflated abdomen phenotype did not develop at any time of adulthood (Fig. 1B-G). Thus, the toxicity of Aβ42 in this non-neuronal cell type can be suppressed by PI3K. These initial observations prompted the analysis of additional cell types.

### Epithelial wing cells are also sensitive to Aβ42 toxicity

**Fig. 2.**
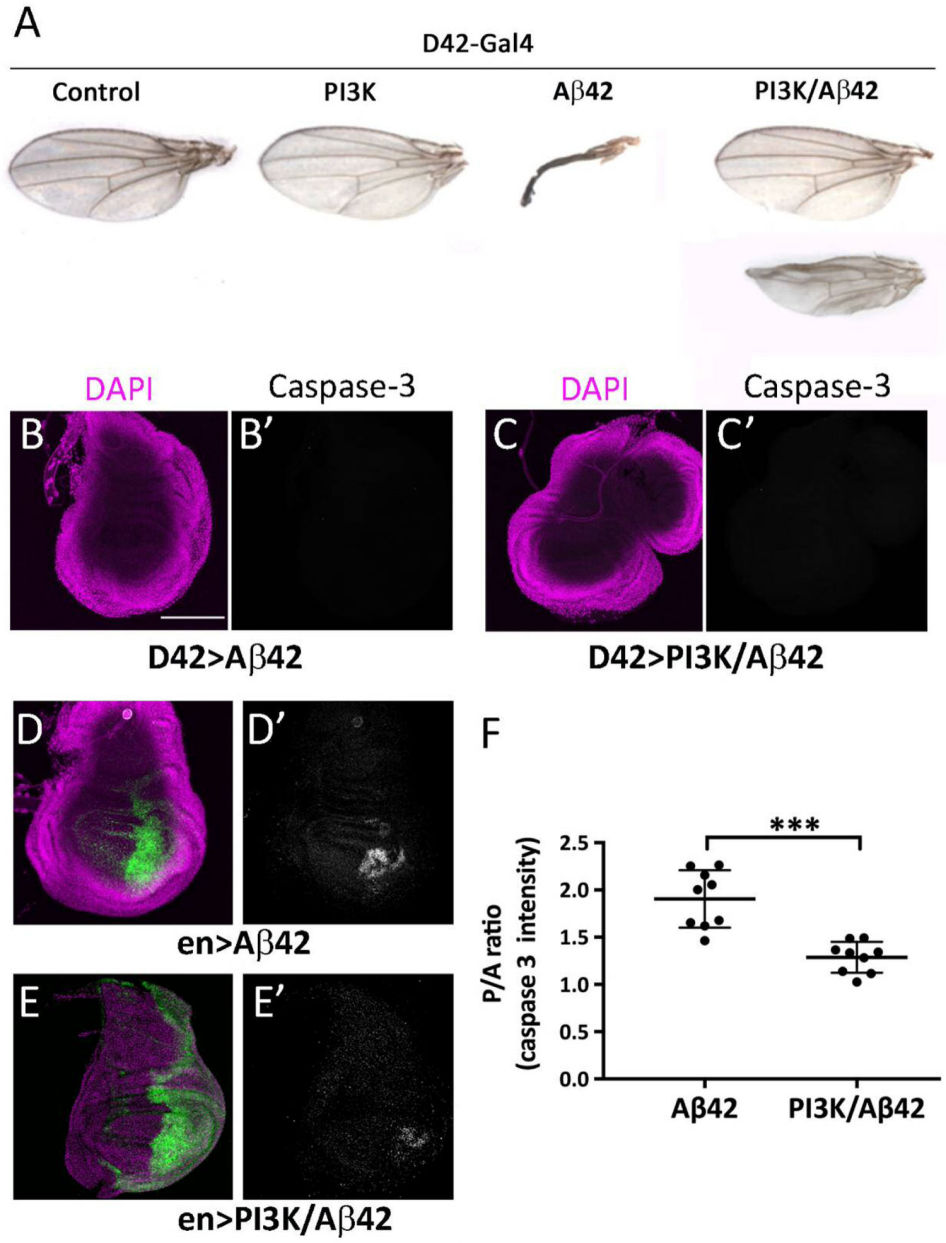
**Wing and apoptotic defects in Aβ42 flies are prevented by PI3K.** (A) Adult wings of genotypes: *UAS-LacZ* (control), *UAS-PI3K^CAAX^, UAS-Aβ42(2x)* and *UAS-PI3K^CAAX^/UAS-Aβ42(2x)* under *D42-Gal4* driver. (B-C′) Same genotypes viewed as imaginal wing discs with no activation of Caspase-3. (D-E′) Same constructs under *engrailed-Gal4/UAS-GFP^nls^* driver (green). Note the active Caspase-3 cells in the driver domain (D′ and E′). (F) Quantification of posterior/anterior, P/A, ratio for Caspase-3 intensities. Bars indicate mean and s.d. Student's *t*-test with ***P<0.001. Scale bar: 100 μm.

The widely used drivers *elav-Gal4* and *D42-Gal4* are considered nervous system specific. However, we demonstrated recently that these two drivers, as well as others, exhibit a transient expression in wing imaginal discs during the first 12 h of development (Casas-Tintó et al., 2017). Taking advantage of this feature (Fig. S2), we searched for deleterious effects of Aβ42 in adult wings to determine if the toxicity in neurosecretory cells is also evident in other cell types early in development. The continuous expression of Aβ42 during development yielded adult wings with severe abnormalities (Fig. 2A). The co-expression of PI3K^CAAX^ suppressed the morphological abnormalities of the adult wings (Fig. 2A). Only 5% of Aβ42/PI3K-expressing adults showed some morphological aberration in their wings and, when present, the defect was reduced (see adult wing in the right panel of Fig. 2A). These findings confirmed the toxicity of Aβ42 in non-neural cells and its suppression by PI3K.

Since the *D42-Gal4* driver is active in wing discs at early stages of development (Casas-Tintó et al., 2017), we questioned if the Aβ42 toxicity could elicit cell death by apoptosis. To that end, we immunostained third instar larval wing discs for activated Caspase-3. No evidence of apoptosis was obtained either in the Aβ42- or in the Aβ42/PI3K-expressing wing discs (Fig. 2B,C). We suspected that the lack of evidences for Caspase-3 activation could be due to the transient expression of *D42-Gal4* in wing discs (first 12 h of development) (Casas-Tintó et al., 2017). Thus, we repeated the experiment using a permanently expressed driver, *engrailed-Gal4* (*en-Gal4*), which is active in the posterior compartment of the wing. In this experiment, the anterior wing disc compartment serves as internal control. Caspase-3 activation was clearly detected in the posterior, by contrast to the anterior, compartment (Fig. 2D). As in previous cases, the co-expression of PI3K significantly reduced the activated Caspase-3 signal (Fig. 2E,F).

### Aβ42 toxicity in epithelial cells alters Wingless signaling

**Fig. 3.**
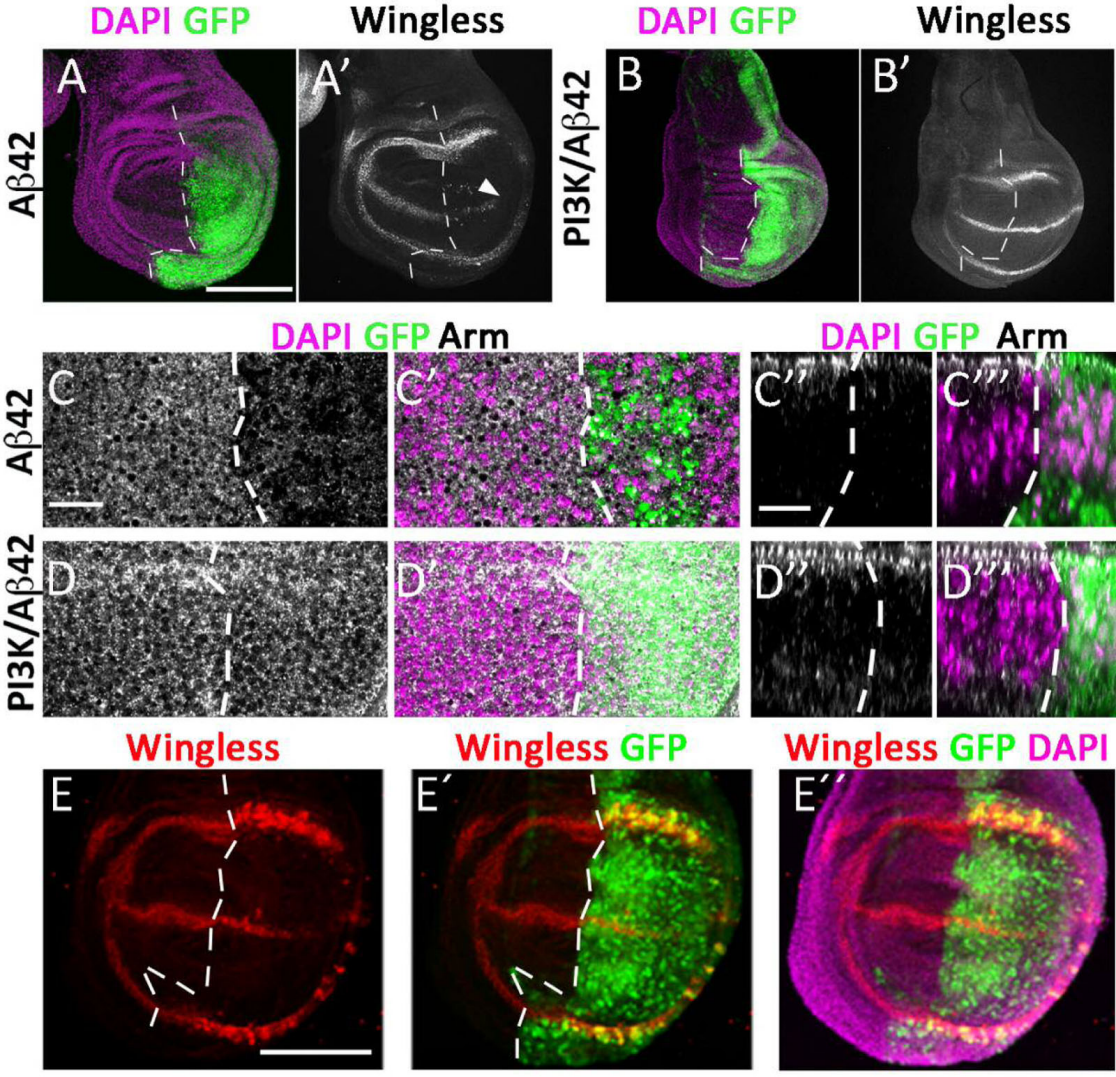
**Aβ42 alters Wingless and Armadillo expression in epithelial wing cells and PI3K suppresses the effect.** Wingless (A-B), Armadillo (C-D) and DAPI (magenta) immunostainings from third instar larval wing discs expressing *UAS-Aβ42(2x)* and *UAS-PI3K^CAAX^/Aβ42(2x)* under *engrailed-Gal4/UAS-GFP^nls^* driver (green). Dotted line separates anterior and posterior wing compartments. Note the loss of Wingless expression (arrowhead in A′) which is restored by PI3K (B′). The same effect is observed for Armadillo (C,C′,D,D′). Orthogonal views of the same discs are shown in C″,C‴,D″ and D‴. The effects persist when the co-expression of p35 prevents Caspase-3 activation and apoptosis (E-E″). Scale bar: 100 μm.

In vertebrates, several Wnt family components are dysregulated in AD (Inestrosa and Varela-Nallar, 2014). Some components have also been implicated in synaptogenesis consistent with the synapse loss observed in the disease (Franciscovich et al., 2008). In view of the toxic effects of human Aβ42 in the fly epithelial cells, we investigated if Aβ42, alone or in conjunction with PI3K, would alter Wingless (*wg*) signaling, a homologue of vertebrate Wnt. To that end, we stained third instar larval wing discs with anti-Wingless (Fig. 3).

The data show a distorted pattern of Wingless expression in the Aβ42-expressing domain (posterior wing compartment, *en-Gal4*) by contrast to the control domain (anterior wing compartment) (Fig. 3A). The distorted expression, however, could be an indirect effect of an aberrant cell shape or cell number, among other factors. To validate the abnormal Wg pattern, we monitored the expression of its functional target, Armadillo (Riggleman et al., 1990; Noordermeer et al., 1994). Armadillo/β-catenin expression is also distorted in the Aβ42-expressing domain (Fig. 3C). Consistent with the experiments above, PI3K suppressed the Wg and Arm alterations caused by Aβ42 (Fig. 3B,D).

The disruption of Wg and Arm expression could be a cause or consequence of the activation of Caspase-3 and cell apoptosis. To sort out the hierarchical order of events, we prevented apoptosis by the co-expression of the baculovirus protein p35 (Hay et al., 1994; Portela et al., 2010). The data show that Aβ42-expressing cells rescued from cell death, exhibit the characteristic abnormality of Wg expression (Fig. 3E). Thus, the disruption of morphogens Wg and Arm are among the early events of Aβ42 toxicity. Interestingly, while the Aβ42-expressing flies in the *D42-Gal4* or *en-Gal4* domains are still able to reach adulthood and exhibit morphological wing abnormalities (see Fig. 2A), flies co-expressing p35 are lethal. This observation is consistent with the strong effects resulting from preventing apoptosis of unfitted cells for which the term ‘undead cells’ was coined (Perez-Garijo et al., 2005, 2009). Actually, it is the contrast between the Wg levels of adjacent cells that triggers cell competition and elimination by apoptosis (Vincent et al., 2011).

In summary, Aβ42 causes toxic effects in non-neuronal cell types, which include early alterations of Wingless and Armadillo signaling, that lead, eventually, to Caspase-3 activation. If the Aβ42-expressing cells are eliminated by apoptosis and the system is still able to proliferate, the organ integrity may be restored. However, if toxic cells are not eliminated, the deleterious effects for the cell system and the organism are more severe.

### Metabolic profiling of Aβ42-expressing flies identifies molecular markers which are suppressed by PI3K

**Fig. 4.**
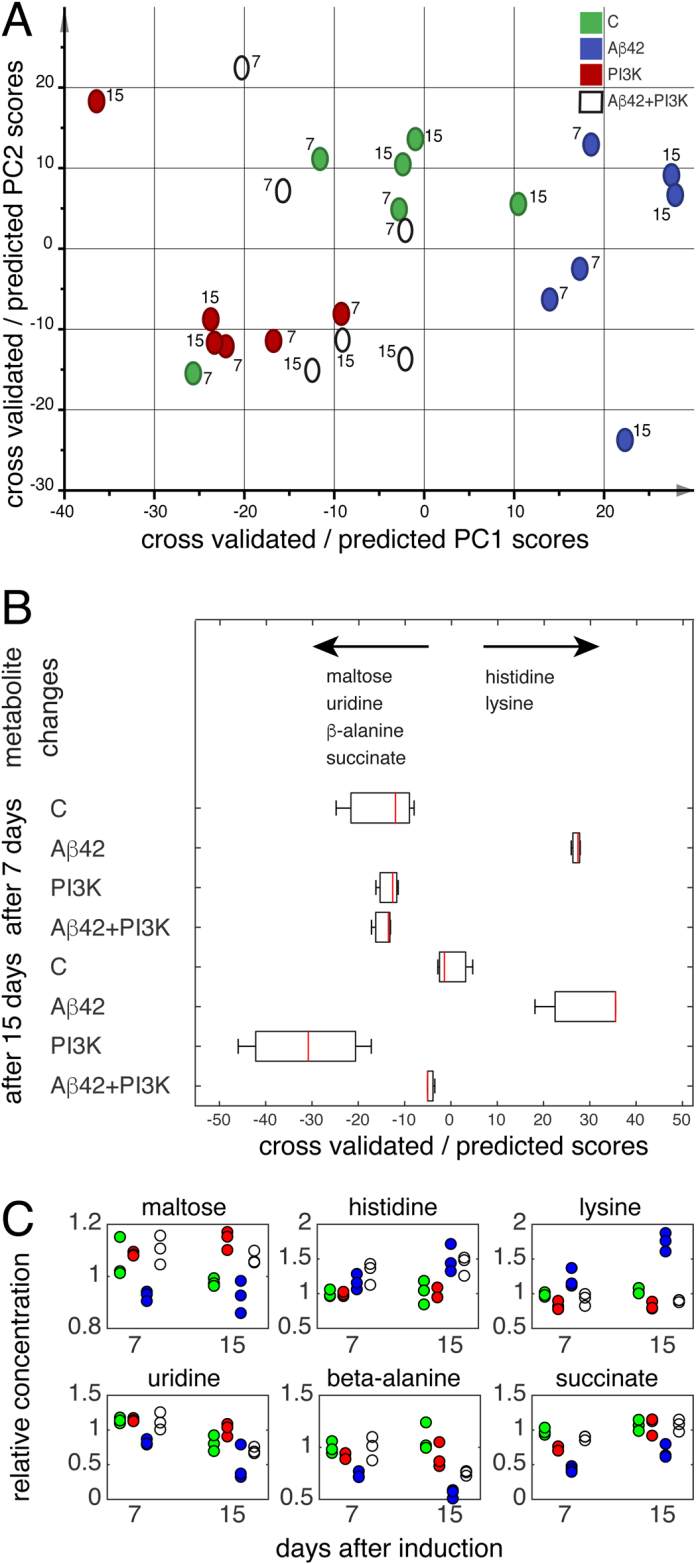
**NMR show a robust metabolite response to high Aβ42 concentrations which is largely reversed by co-expression of PI3K.** (A) O2PLS-DA model separating metabolite profiles of control, Aβ42- and PI3K-expressing flies. The cross-validated scores of control (green), Aβ42- (blue) and PI3K-expressing (red) flies show a robust separation of the three groups. The scores of Aβ42+PI3K-expressing flies were predicted based on this model. The values show that co-expression of PI3K reverts the metabolic effects of Aβ42. Metabolite variations along the x-axis include increases in glucose, histidine, uracil, phosphocholine and lysine, and decreases in maltose, uridine, succinate and β-alanine, and variations along the y-axis include increases in arginine and β-alanine. (B) OPLS-DA model separating metabolite profiles of Aβ42 from control and PI3K-expressing flies. There is a clear separation of the cross-validated scores. The scores of Aβ42+PI3K flies are similar to control and PI3K. Thus, PI3K prevents the metabolic effects of Aβ42 at day 7 and 15. Metabolite differences are indicated under the scores plot. Box and whisker plots indicate the median, the first and third quartiles, and the minimum and maximum score values. (C) Relative metabolite concentrations. Colors are as in A. Concentrations were normalized to the median in control flies.

Fly models of AD show changes in the metabolite profile both in nervous and non-nervous tissues (Ott et al., 2016). Thus, we explored the metabolome of adult flies expressing high levels of Aβ42, either alone or in combination with PI3K. Expression was triggered at day 0-3 post eclosion in order to avoid potential effects during larval development. The metabolite profile was monitored by NMR 7 and 15 days later (i.e. day 7-10 or 15-18 of adulthood; Fig. 4). In order to focus on non-neuronal tissues, we studied the metabolite response of abdomens. In this body part, the *elav-Gal4* driver shows temporal expression as demonstrated by the GTRACE procedure (Fig. S3).

We used an O2PLS-DA model to visualize the effects of Aβ42 and PI3K expression (Fig. 4A). The cross-validated scores show a robust separation from the control (green), and those submitted to Aβ42 (blue) and PI3K expression (red) (Fig. 4A). Metabolite variations along the x-axis include increases in glucose (3.89, 3.47, 3.45, 3.39, 3.23 ppm), histidine (7.78, 7.05, 3.12 ppm), uracil (7.53, 5.79 ppm), phosphocholine (4.15, 3.21 ppm) and lysine (3.01, 1.88, 1.72, 1.44 ppm), and decreases in maltose (5.40, 3.94, 3.90, 3.85, 3.68, 3.61, 3.27 ppm), uridine (7.85, 5.89 ppm), succinate (2.39 ppm) and β-alanine (3.16, 2.54 ppm). Variations along the *y*-axis include increases in arginine (1.92, 1.64 ppm) and β-alanine. The scores of the Aβ42+PI3K-expressing flies were then calculated. As seen in Fig. 4A, the values fall between the control and PI3K scores and show no overlap with those of Aβ42, indicating that co-expression of PI3K essentially reverts the metabolic effects of Aβ42.

To further analyze the effect of Aβ42-expression, and that of its co-expression with PI3K, an OPLS-DA model was made to separate Aβ42 flies from both control and PI3K-expressing flies (Fig. 4B). Aβ42-expressing flies had higher levels of histidine and lysine and lower levels of maltose, uridine, β-alanine and succinate (Fig. 4B). The predicted scores of the Aβ42+PI3K-expressing flies also show a reversion of the effect of Aβ42-expression.

To compare the effects of Aβ42 and PI3K expression, OPLS-DA models were also made for PI3K expression versus control and Aβ42 expression, and for Aβ42+PI3K expression versus control and PI3K expression (Table S1). PI3K-expressing flies were also different from control and Aβ42-expressing flies and showed higher levels of maltose and lower levels of histidine, lysine and potentially arginine. In contrast, Aβ42+PI3K-expressing flies were not different from control and PI3K-expressing flies (Table S1). Finally, we examined the variation in concentration of the affected metabolites as a function of time and expressed proteins. Relative concentrations of maltose, histidine, lysine, uridine, β-alanine and succinate are shown in Fig. 4C. Notably, all Aβ42-induced metabolite changes except the increase in histidine were reversed by PI3K.

It is widely accepted that neurons produce the majority of Aβ in an activity-dependent manner (Kamenetz et al., 2003). However, several studies have demonstrated that glial cells also play important roles in AD pathology (De Strooper and Karran, 2016). Actually, AD patients often die by bronchopneumonia and cardiovascular diseases (Brunnström and Englund, 2009; Attems et al., 2005) rather than by neuronal loss. These facts argue that Aβ toxicity in non-neuronal tissues could contribute to the eventual death of the patients, and justified our evaluation of Aβ42 in neurosecretory and epithelial tissues in search for an early diagnosis.

In leukokinin cells, Aβ42 generates an inflated abdomen akin to that described in *Drosophila* renal failure models (Denholm, 2013). Leucokinin, analog of mammalian vasopressin, regulates Malpighian tubule fluid secretion, diuresis, fluid balance (Terhzaz et al., 1999; Chen et al., 1994) and cardio and respiratory regulation in insects (Cantera and Nässel, 1992). Aβ42 toxicity in neurosecretory cells cannot be related to synapses because these cells lack synapsin, subsynaptic reticulum and synaptic specializations altogether (Landgraf et al., 2003). Peptide secretion uses mechanisms different from those for neurotransmitter release in synapses (Mansvelder and Kits, 2000). The toxicity of Aβ42 in epithelial cells points towards a more basic reason which, eventually, leads to activation of Caspase-3. One of these basic mechanisms seems to be defective Wg signaling.

The metabolite variations include increases in glucose, histidine, uracil, phosphocholine and lysine in Aβ42 and increases in maltose, uridine, succinate and β-alanine in PI3K flies (Peterson et al., 1998). Interestingly, uracil is metabolically connected to uridine and β-alanine by one and three enzymatic steps, respectively. Both equilibria are shifted towards uracil in Aβ42 flies. Aβ42-expressing flies had higher levels of histidine and lysine and lower levels of maltose, uridine, β-alanine and succinate (Fig. 4B) than control and PI3K flies. Histidine is a precursor for histamine and carnosine biosynthesis, and a powerful antioxidant and anti-inflammatory factor (Peterson et al., 1998). Increased histidine levels were observed in response to acute stress and artificial selection for stress resistance in *Drosophila* (Malmendal et al., 2013; Overgaard et al., 2007). Histidine has earlier been shown to increase in *Drosophila* expressing both benign and toxic Aβ (Ott et al., 2016), which is very similar to the present system where histidine increased in Aβ42 flies, independent of co-expression of PI3K. In humans, high histidine was found in urine from patients with Parkinson's disease (Luan et al., 2015), and additional high levels of lysine were found in plasma from patients with mild cognitive impairment (Trushina et al., 2013).

Maltose has been suggested to have a chaperone-like function (Kaplan and Guy, 2004; Pereira and Hünenberger, 2006) and increased levels were observed in *Drosophila* expressing non-toxic levels of Aβ40 and Aβ42 and in short and long-term stress responses (Ott et al., 2016; Malmendal et al., 2013; Overgaard et al., 2007). Uridine is precursor of phosphatidylcholine, a major component of cellular membranes, and a reduction in cerebrum-spinal fluid (CSF) of AD patients was suggested to be linked to neuronal deficits (Czech et al., 2012). β-alanine is related to inhibitory neurotransmitters (Mori et al., 2001). Succinate serves as an electron donor to the transport chain in the citric acid cycle and lower levels were found in CSF of AD patients (Redjems-Bennani et al., 1998) and in all brain regions in an AD mouse model (Salek et al., 2010).

The Aβ42-induced metabolite changes agree with those caused by toxic Arctic Aβ42 (E22G) relative to non-toxic levels of Aβ42 and Aβ40 in abdomens (Ott et al., 2016) in that we see an increase in histidine and a decrease in maltose in both systems (Fig. 4B,C). However, we do not detect increases in gluconic acid and a potentially hydroxylated aromatic compound. This suggests a somewhat different response in the highly expressing Aβ42 flies or, more general, specificity in the metabolic signature caused by different Aβ peptides (Grochowska et al., 2017).

## MATERIALS AND METHODS

### Fly strains

The following strains were obtained from the Bloomington Stock Center (NIH P40OD018537) (http://flystocks.bio.indiana.edu/): *elav^c155^-Gal4*, BL-458 (Lin and Goodman, 1994); *D42-Gal4*, BL-8816 (Chan et al., 2002); *engrailed-Gal4*, BL-1973 (Lawrence et al., 1995); *Tubulin-Gal80^TS^*, BL-7019 (McGuire et al., 2003); *UAS-LacZ*, BL-1776 (Brand and Perrimon, 1993); *UAS-PI3K^CAAX^* BL-8294 (Parrish et al., 2009) and *UAS-G-TRACE* BL-28280 (Evans et al., 2009). In addition, we obtained the lines: *leucokinin^M7^-Gal4* from Dr F. Benjumea (Center for Molecular Biology, Madrid, Spain) (de Haro et al., 2010) and *UAS-Aβ42(2x)* from Dr P. Fernández-Fúnez (University of Florida, USA) (Casas-Tintó et al., 2011). The *UAS-Aβ42(2x)* construct contains two copies of the gene encoding the human *Aβ42* peptide which has proven effective to cause β-amyloid deposits immune-positive for 6E10 antibody (Covance catalog # SIG-39320).

### Activation of the Gal4/UAS system and sample preparation

Flies of genotypes containing the Gal4/UAS/Gal80^TS^ constructs were grown at 17°C, and transferred to 29°C as 0–3-day-old adults to allow the expression of the Gal4. The three days of adulthood prior to the onset of Gal4 expression were required to allow maturation of adult neural structures. Flies of the desired age with the Gal4 system activated (see diagram in Fig. 1) were collected, rapidly frozen in liquid nitrogen and stored at −80°C until the required amount was obtained. Using a sieve of appropriate size, thorax-abdomen samples were obtained and used for further processing under NMR (see below).

### Immunostaining

Third instar larval tissues and adult brains were dissected and fixed with 4% formaldehyde in phosphate-buffered saline for 20 min, washed three times with PBS1×0.1% Triton-X, and mounted in Vectashield medium with DAPI, or incubated with primary and secondary antibodies. The following antibodies and dilutions were used: anti-Wingless mouse 1:20 (DSHB); anti-Activated caspase-3 IHC rabbit 1:100 (Cell Signal) and anti-Armadillo mouse 1:50 (DSHB). Preparations were imaged in a Leica SP5 confocal microscope and images were processed by ImageJ (NIH).

### Sample preparation for NMR

For each condition we prepared three replicates of 20 pooled fly thorax-abdomens for NMR spectroscopy. Samples were directly lyophilized and stored at 4°C (Jensen et al., 2013). Materials were mechanically homogenized with a TissueLyzer (Quiagen) in 1 ml of ice-cold acetonitrile (50%) for 3×1 min. Hereafter samples were centrifuged (10,000 ***g***) for 10 min at 4°C and the supernatant (900 μl) was transferred to new tubes, snap frozen and stored at −80°C. The supernatant was then lyophilized and stored at −80°C. Immediately before NMR measurements, samples were rehydrated in 200 ml of 50 mM phosphate buffer (pH 7.4) in D_2_O, and 180 ml was transferred to 3 mm NMR tubes. The buffer contained 50 mg/l of the chemical shift reference 3-(trimethylsilyl)-propionic acid-D4, sodium salt (TSP), and 50 mg/l of sodium azide to prevent bacterial growth.

### NMR experiments

NMR measurements were performed at 25°C on a Bruker Avance III HD 800 spectrometer (Bruker Biospin, Rheinstetten, Germany), operating at a ^1^H frequency of 799.87 MHz, equipped with a 3 mm TCI cold probe. ^1^H NMR spectra were acquired using a single 90°-pulse experiment with a Carr-Purcell-Meiboom-Gill (CPMG) delay added, in order to attenuate broad signals from high-molecular-weight components. The total CPMG delay was 194 ms and the spin-echo delay was 4 ms. The water signal was suppressed by excitation sculpting, potentially masking changes in metabolites (mostly sugar units) resonating in this region. A total of 128 transients of 32 K data points spanning a spectral width of 20 ppm were collected, corresponding to a total experimental time of 6.5 min.

### NMR data and analyses

The spectra were processed using iNMR (www.inmr.net). An exponential line-broadening of 0.5 Hz was applied to the free-induction decay prior to Fourier transformation. All spectra were referenced to the TSP signal at −0.017 ppm, automatically phased and baseline corrected. The spectra were aligned using *i*coshift (Savorani et al., 2010). The spectra were normalized to total intensity in order to suppress separation based on variations in amount of sample. Metabolite assignments were done based on chemical shifts only, using earlier assignments and spectral databases previously described (Bertram et al., 2006; Cui et al., 2008; Malmendal et al., 2006; Ott et al., 2016), and comparison with *Drosophila* metabolites identified by mass spectrometry (Chintapalli et al., 2013). NMR spectra with the resulting assigned metabolites are provided in Fig. S1.

Although unsupervised methods like principal component analysis (PCA) are highly informative when analyzing differences in metabolite profiles, other effects may hide the variation of interest. Thus, orthogonal projection to latent structures discriminant analysis (O2PLS-DA) (Trygg and Wold, 2003) was used to focus the analysis on the variation that separates the different combinations of protein expression and age. The O2PLS-DA models were carried out on Pareto-scaled data. They were validated by cross validation, where randomly chosen groups of samples were left out to predict group membership for the excluded samples, until predicted values had been obtained for all samples.

O2PLS-DA was used to make a model separating 7−10 and 15−18-day-old control, Aβ42 and PI3K in to three groups. Similarly, OPLS-DA was used to make a model separating Aβ42 from control and PI3K in 7−10 and 15−18-day-old flies together. In both cases the relative similarity of the Aβ42+PI3K flies to the separated groups was estimated. Models for PI3K versus control and Aβ42 and Aβ42+PI3K versus control and PI3K were also made for 7−10 and 15−18-day-old flies together.

We also tested which metabolites correlated with the overall between group differences in the metabolome. Significant spectral correlations were identified by applying sequential Bonferroni correction (*P*<0.05) for an assumed total number of 100 metabolites.

## Supplementary Material

Supplementary information
