# Supplementary material for: Amyloid β42 peptide is toxic to non-neural cells in Drosophila yielding a characteristic metabolite profile and the effect can be suppressed by PI3K
Source: Biol Open. 2017 Nov 15;6(11):1664–71. doi: 10.1242/bio.029991 (PMC5703620; doi:10.1242/bio.029991)
Supplement: Supplementary information [file biolopen-6-029991-s1.pdf]

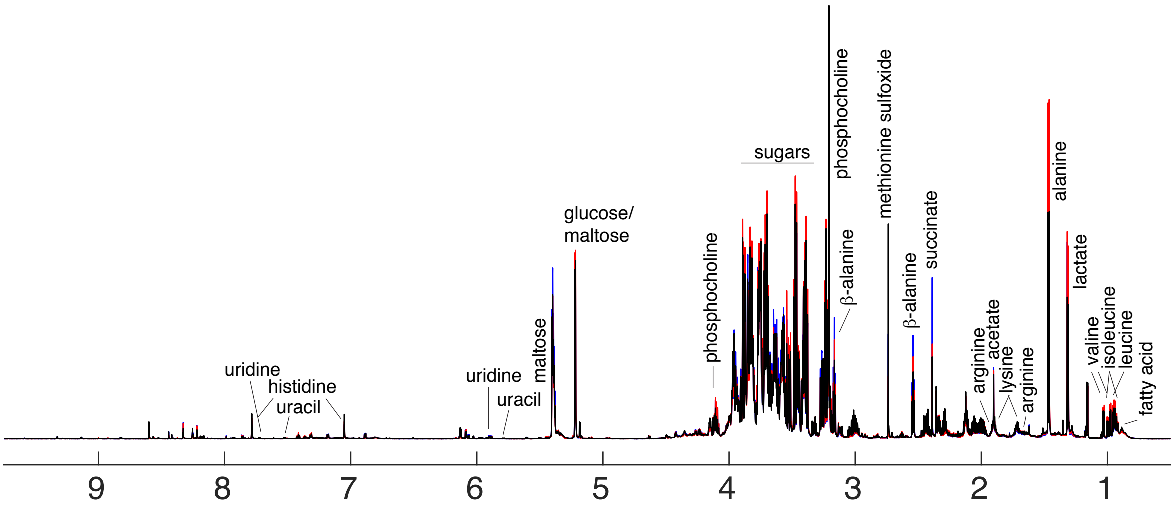

Fig. S1.- ^{1}H nuclear magnetic resonance (NMR) spectra of *Drosophila melanogaster* metabolites. NMR spectra of flies expressing PI3K (blue), Aβ42 (red) and both Aβ42 and PI3K (black) 15 days after induction of protein expression. The displayed spectra show the mean intensities of three replicates.

Biology Open • Supplementary information

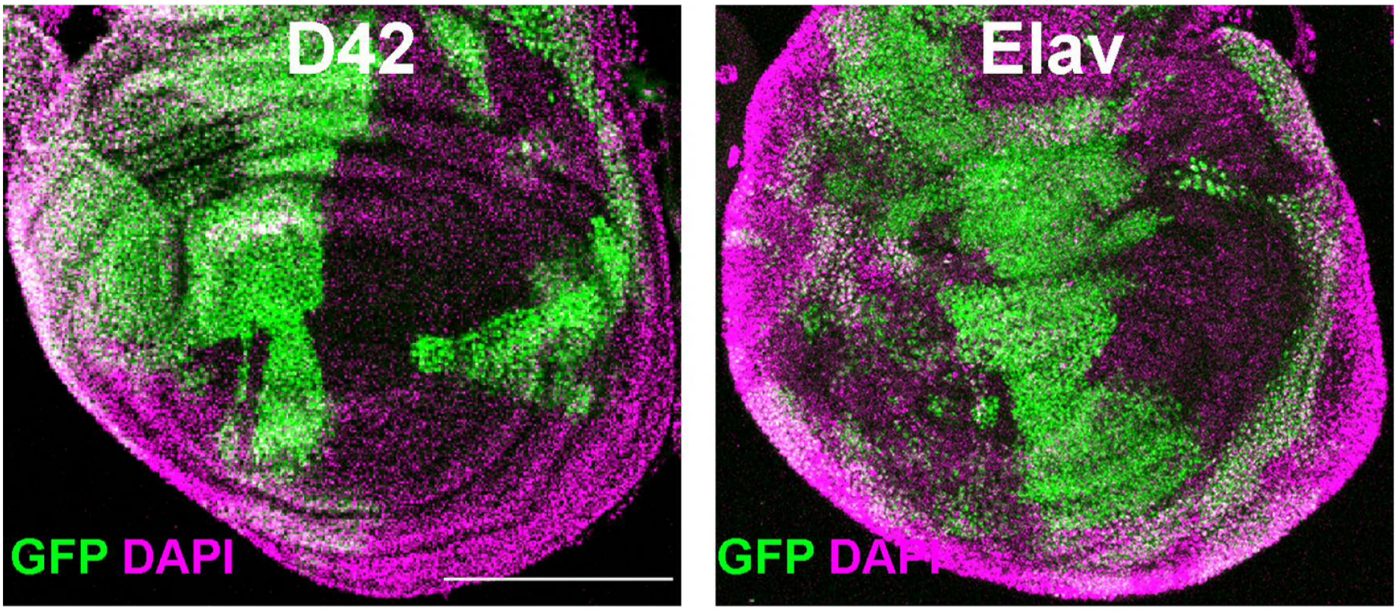

Suppl Fig. S2

Fig. S2.- Transient activation of Gal4 drivers in the wing disc. Using the G-TRACE technique, the initial activation of enhancers controlling the Gal4 expression can be monitored by GFP. Once activated, the GFP reporter maintains its expression in a Gal4 independent manner allowing its visualization even when the enhancer controlling the Gal4 expression is switched off. The images shown correspond to late third instar larva, a time at which the *D42* and *elav-Gal4* drivers are no longer expressed in the wing disc.

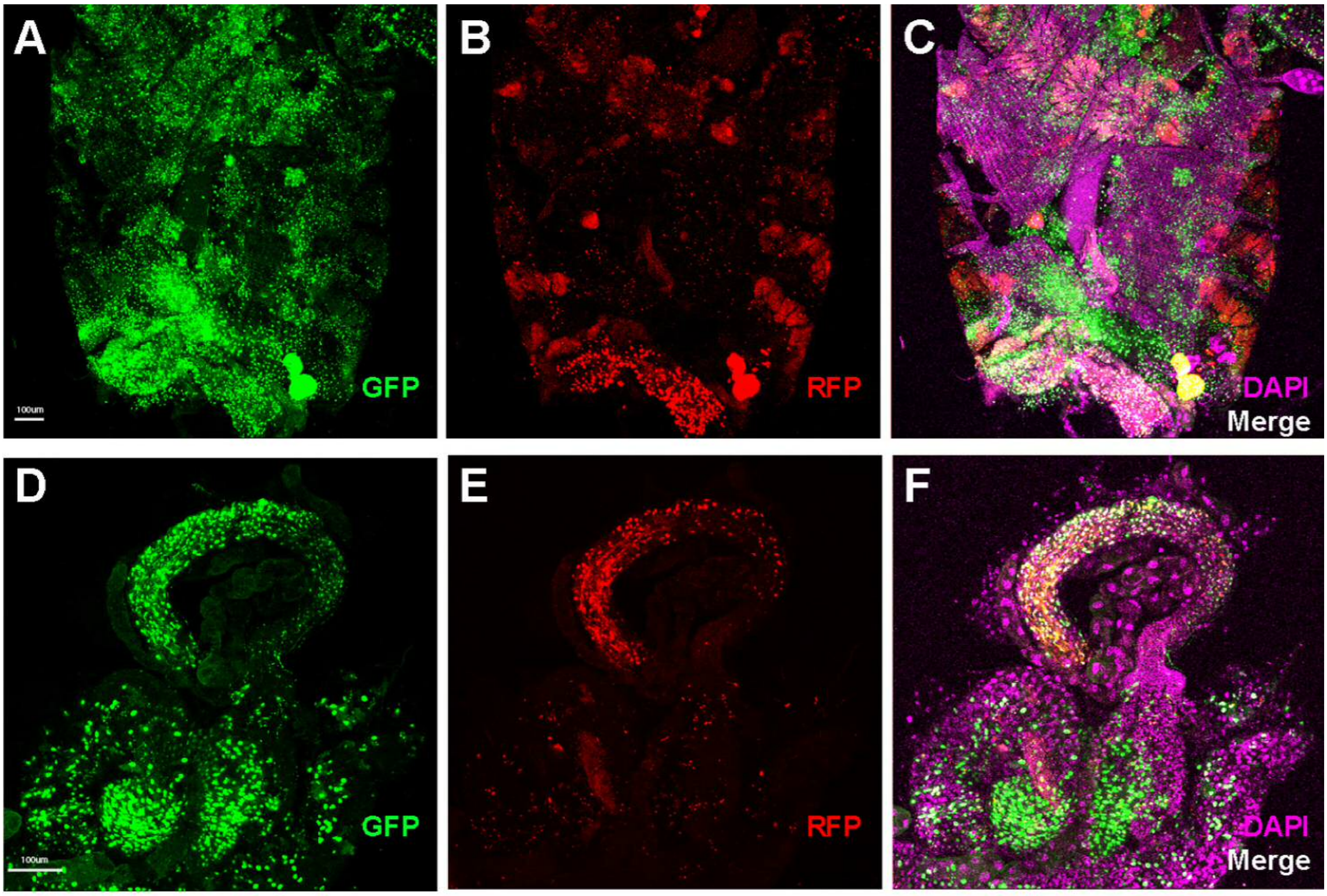

Supp. Fig. S3

Fig. S3.- Transient activation of *elav-Gal4* in the adult. Images show a squashed adult female abdomen **(A-C)** and thorax **(D-F)** 15-days post switching-on the driver. Genotype: *elav-Gal4/+ ; UAS-RedStinger, UAS-FLP, Ubip63FRT-stop-FRT-Stinger/+ ; tub-Gal80*^{TS}/+. The historical (green) versus the current (red) expression domains of the driver are revealed. All these cells contributed to the metabolic signature of Aβ42.

Biology Open • Supplementary information

Table S1.

O2PLS-DA and OPLS-DA model statistics for parameter prediction from metabolite data

**Modeled groups**	**Age** (days)	**A**[†]	**N**[‡]	$\mathbf{R^{2§}}$	$\mathbf{Q^{2*}}$
control *vs.* Aβ42 *vs.* PI3K	7, 15	2+0	18	0.40	**0.41**
Aβ42 *vs.* control and PI3K	7, 15	1+1	18	0.42	**0.76**
	7	1+0	9	0.46	**0.81**
	15	1+0	9	0.39	**0.70**
PI3K *vs.* control and Aβ42	7, 15	1+1	18	0.42	**0.64**
Aβ42+PI3K *vs.* control and PI3K	7, 15	1+1	18	0.52	**-0.11**

[†]A describes the number of model components where the first number accounts for the predictive component(s) correlating with the predicted variable, and the second the orthogonal component(s).

[‡]N describes the number of observations included in the model.

[§]R^2 describes how much of the total metabolite variation that is explained by the model.

[*]Q^2 represents the predictability of the total model and is related to the statistical validity of the model. Q^2 was calculated using cross-validation with all measurements for one condition left out at a time.

Biology Open • Supplementary information
